# Influence of horizontal resistance loads on spatiotemporal and ground reaction force variables during maximal sprint acceleration

**DOI:** 10.1371/journal.pone.0295758

**Published:** 2023-12-12

**Authors:** Norihide Sugisaki, Kai Kobayashi, Takaya Yoshimoto, Naotoshi Mitsukawa, Hiroyasu Tsuchie, Yohei Takai, Hiroaki Kanehisa

**Affiliations:** 1 Center for Liberal Arts, Meiji Gakuin University, Yokohama, Kanagawa, Japan; 2 Faculty of Information Sciences and Arts, Toyo University, Kawagoe, Saitama, Japan; 3 Faculty of Welfare Society, The International University of Kagoshima, Kagoshima, Kagoshima, Japan; 4 Faculty of Human Sciences, Toyo Gakuen University, Bunkyo-ku, Tokyo, Japan; 5 Faculty of Law, Toyo University, Bunkyo-ku, Tokyo, Japan; 6 Faculty of Sports and Life Science, National Institute of Fitness and Sports in Kanoya, Kanoya, Kagoshima, Japan; 7 National Institute of Fitness and Sports in Kanoya, Kanoya, Kagoshima, Japan; University of Belgrade: Univerzitet u Beogradu, SERBIA

## Abstract

This study aimed to elucidate the influence of horizontal resistance loads on the spatiotemporal and ground reaction force (GRF) variables during maximal sprint acceleration. Nine male sprinters (20.2 ± 1.2 years; 175.3 ± 4.5 cm, 69.7 ± 6.1 kg) performed sprint-running with six loading conditions of one unresisted and five resisted loads of 4, 6, 8, 10, and 12 kg using a resistance training device with intelligent drag technology. During the trials, the GRFs for all steps were determined using a 50-m force plate system. The spatiotemporal and GRF variables at running velocity of every 0.5 m/s were obtained and compared across the loading conditions. The maximal running velocity under 0, 4, 6, 8, 10, and 12 kg loading conditions were 9.84 ± 0.41, 8.55 ± 0.41, 8.09 ± 0.33, 7.62 ± 0.34, 7.11 ± 0.31, and 6.71 ± 0.29 m/s, respectively. ANOVA revealed significant main effects of load on the measured variables (*η*^2^ = 0.236–0.715, *p* < 0.05), except for stance-averaged anteroposterior GRF and braking impulse. However, the observed differences between the loading conditions were small, with approximately 4% (1.3–7.5%) for the GRF variables and approximately 9% (1.2–22.3%) for the spatiotemporal variables. The present study indicates that horizontal resistance load in sprint acceleration has little impact on the spatiotemporal and GRF variables at a given running velocity. In contrast to a general recommendation, one should adopt a heavy load in resisted sprint aiming to improve performance in the earlier stage of maximal sprint acceleration.

## Introduction

Sprint running with horizontal resistance loading (i.e., resisted sprint) has been used as a modality to improve sprint acceleration performance [1,2]. For the implementation of this approach, it has been recommended to use a relatively light load (< 10–20% of the body mass in sled towing or ~10% velocity loss) [1,3,4] because heavy loads cause significant changes (~20% or more) in sprint mechanics, such as longer ground contact time, decreased step length (SL), anterior inclination, and greater application of horizontal force and impulse to the ground [3–6]. However, a recent study reported that a nine-week resisted sprint training program using a heavy load, yielding a 50% velocity loss, improved sprint performance without changes in sprint kinematics [7]. Contrary to the previous recommendations, this report suggests the effectiveness of using heavy loads in resisted sprint training.

In most previous studies that investigated the differences in sprint kinematics or kinetics between unresisted and resisted conditions, the corresponding variables were obtained at a given distance or step number from the starting point [3–8]. However, in the maximal resisted sprint, the running velocity at a given distance or step number differs according to the loads adopted; that is, the heavier the load, the lower the running velocity at a given distance or step number. For example, the take-off velocities of the second step in sled towing with loads equivalent to 12.6 and 32.2% of the body mass were 91.2% (5.2 m/s) and 77.2% (4.4 m/s), respectively, compared to the unresisted sprint (5.7 m/s) [4]. Therefore, comparisons at a given distance or step number include the influence of differences in running velocity. During maximal unresisted sprint acceleration, especially in the initial phase, sprint mechanics change dramatically with running velocity. In the initial acceleration phase, an increase in running velocity of 1 m/s is accompanied by an increase in SL by ~20%, net anteroposterior ground reaction impulse by ~13%, step-averaged anteroposterior ground reaction force (*F*_ap_) by ~18%, decreases in ground contact time by ~10%, and trunk inclination by ~6 deg [9–11]. These findings suggest that previous reports on the alteration in sprint mechanics during resisted sprint [1,3–6,8] may have been influenced by the running velocity at which the kinetic and kinematic variables have been analyzed. Thus, there should be no differences in sprint mechanics between the loading conditions if comparisons were made at a given running velocity.

With regard to ground reaction forces (GRFs) at a given velocity during resisted sprints, three studies have already attempted to examine the influences of loading conditions on *F*_ap_–velocity relationships [12–14]; however, the findings remain controversial. One study reported that when *F*_ap_ at a given running velocity was adopted, the *F*_ap_-velocity relationship derived from the resisted sprint was similar to that for the corresponding phase during an unresisted sprint [12]. This supports the assumption that sprint mechanics do not differ between loading conditions when the corresponding variables are compared at a given velocity. However, others have shown that as the load increases, the slope of the *F*_ap_–velocity relationship becomes more negative [14] or positive [13]. Thus, the influence of resistance loads on sprint acceleration kinetics has not yet been elucidated.

This study aimed to clarify the influence of resistance loads on the spatiotemporal and GRF variables during sprint acceleration. To this end, we obtained the GRF data for all steps in unresisted and resisted sprints with a wide range of loads from moderate to very heavy using a 50-m force plate system [10,15–17], and examined the differences between loading conditions in the spatiotemporal and GRF variables that were analyzed at a given running velocity. We hypothesized that there would be no significant inter-load differences in the values of the spatiotemporal or GRF variables when compared at a given running velocity.

## Materials and methods

### Subjects

The recruitment period for this study was from 21 October 2020 to 31 March 2023. The subjects were limited to sprinters who have experienced resisted sprinting to ensure the stability and reproducibility of resisted and unresisted sprint motions. Nine male collegiate sprint runners participated in this study (20.2 ± 1.2 years; 175.3 ± 4.5 cm, 69.7 ± 6.1 kg; mean ± SD). Eight specialized in 100 m (personal best: 10.57 ± 0.34 s) and one in 200 and 400 m (personal best for 200 m: 22.32 s). The subjects have been competing in sprint events for 7.6 ± 1.4 years. This study was approved by the Ethics Committee of the National Institute of Fitness and Sports in Kanoya (#5–60), and all procedures were conducted in accordance with the Declaration of Helsinki. Prior to the experiments, all subjects were fully informed of the purpose and risks of the experiment and provided written consent.

### Procedures

Prior to the test session, the subjects performed warm-up exercises, including ~8 min of running, ~10 min of static and dynamic stretching, ~10 min of running technique exercises, 2–4 submaximal-to-maximal sprinting, and five resisted sprints under the same conditions as the test trials. Following at least 5 min of rest after the warm-up session, the subjects performed one unresisted and five resisted sprints with different loading conditions using a resistance training device with intelligent drag technology (1080 Sprint, 1080 Motion, Sweden) from a standing split-stance position. This device was developed as a portable resistance training device featuring a servo motor (2000 RPM OMRON G5 Series Motor, OMRON Corporation, Kyoto, Japan) and has been used in recent resisted sprint studies [13,18–21]. In resisted sprint, “normal mass resistance mode” was used, simulating the inertial properties of a normal mass (i.e., a cable-driven weight stack) in gravity (https://1080motion.com/). The load settings of the device, which simulated inertial mass for the five resisted sprints, were 4, 6, 8, 10, and 12 kg. These loads were determined in preliminary experiments to include moderate (10–14% velocity loss) to very heavy loads (>30% velocity loss), following previous studies reporting the influence of horizontal loading on sprint mechanics [3–6]. The subjects wore their own spikes. All test trials were performed on an indoor track with a series of 54 force plates (TF-90100, TF-3055, and TF-32120; Tec Gihan, Uji, Japan) embedded [10,15–17]. Four of the force plates were for measuring GRFs of both hands and feet during the crouched start. Therefore, the remaining 50 force plates were used in this experiment. The temperature within the facility was controlled by an air conditioning system and there was no wind. The temperature and humidity during the experiment were 18–20°C and 65–70%, respectively. The 1080 Sprint device was placed approximately 3 m behind the starting line and the cord from the motor was held at the waist of the runner using a belt. It has been shown that performing resisted sprint prior to unresisted sprint or performing heavier resisted sprint before lighter resisted sprint affects the motor pattern of the subsequent trial and makes the movement to be unnatural [6]. In the present study, therefore, the subjects performed one trial per loading condition in ascending order of load to avoid fatigue and/or changes in the motor pattern. An additional trial was conducted when the subjects or their coaches felt that they had failed to achieve their best performance. The sprinting distance was 60 m for the unresisted sprint and 50, 45, 40, 35, and 30 m for the 4, 6, 8, 10, and 12 kg resisted sprints, respectively. The distance in each loading condition was determined with the two criteria: reaching to maximum running velocity and minimizing fatigue. The consistency with the criteria was confirmed in preliminary experiments. Each subsequent trial was performed only after the subject had rested more than 5 minutes and declared full recovery.

### Data analyses

**Fig 1:**
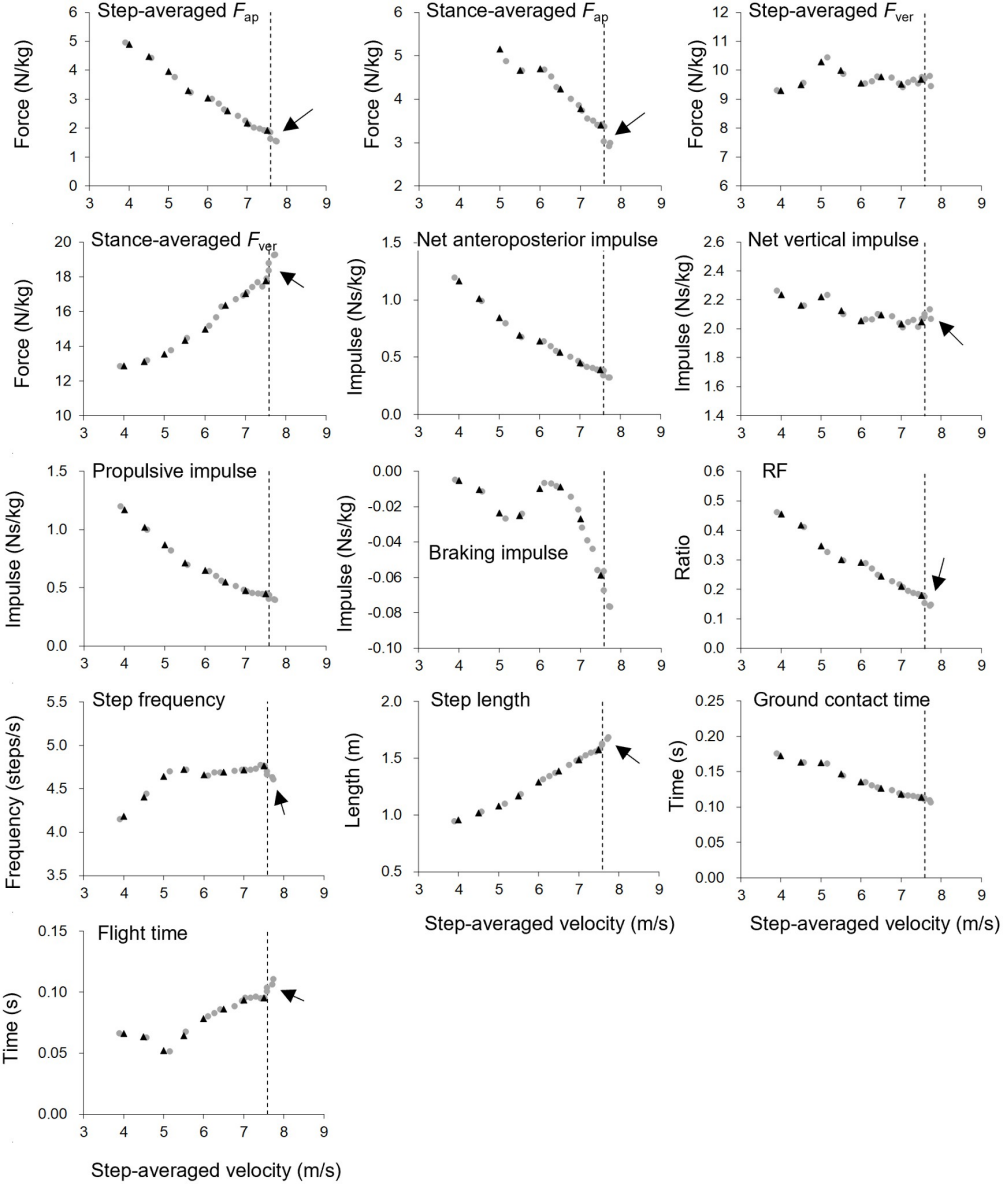
Example of each measurement variable plotted against step-averaged velocity. Data are from one subject in 6 kg loading condition. Gray circles: Measured values. Black triangles: Values at every 0.5 m/s obtained using spline interpolation technique. A few subjects showed the rapid change in some variable near the maximal step-averaged velocity in resisted sprint (arrows) into “bouncing” manner. Therefore, steps at less than 98% maximal step-averaged velocity in each trial (vertical dashed line) were used for analysis. *F*_ap_: Anteroposterior ground reaction force. *F*_ver_: Vertical ground reaction force. RF: Ratio of stance-averaged *F*_ap_ to resultant ground reaction force.

Each force plate can measure three axes (anteroposterior, mediolateral, and vertical) of force and moment. In this study, we used anteroposterior and vertical force signals. The analog force signals of each force plate were collected in a control box (FP Control Unit, Tec Gihan, Uji, Japan), analog-to-digital converted, and sent to a computer. The 50 force plates were treated as one unit (a 50-m long force plate) using a dedicated software, and *F*_ap_, vertical GRF (*F*_ver_), and the location of center of pressure on the ground during ground contact were calculated at a sampling rate of 1000 Hz. The force data were filtered using a fourth-order zero-lag low-pass Butterworth filter with a cutoff of 50 Hz [10,22]. The magnitude of the resultant GRF (*F*_res_) was calculated as the vector sum of *F*_ap_ and *F*_ver_. The threshold of ground contact and take-off was set at 20 N for *F*_ver_ [10,22]. The SL, step frequency (SF), and step-averaged velocity were calculated in accordance with the procedures described in a previous study [17]. SL was calculated as the distance between the location of the center of pressure on the ground at the takeoff of one step and that of the following step. The SF was calculated as the inverse of step duration. The step-averaged velocity was calculated by multiplying SF by SL. In addition, the following spatiotemporal and GRF variables were calculated: ground contact and flight time, step-averaged *F*_ap_ and *F*_ver_, and stance-averaged *F*_ap_ and *F*_ver_. The ratio of *F*_ap_ to *F*_res_ for each ground contact (RF), being an index of force application technique and a determinant factor of sprint performance, was determined by dividing the stance-averaged *F*_ap_ by the stance-averaged *F*_res_ [23,24]. The net anteroposterior and vertical impulses for each step were calculated using time integrations of *F*_ap_ and *F*_ver_, respectively. The propulsive and braking impulses for each step were calculated using the time integration of the positive and negative *F*_ap_, respectively. To eliminate variability owing to bilateral differences, the time-course data for each variable were smoothed using a two-step moving average. In resisted sprint, three subjects abruptly changed their sprint mechanics into “bouncing” manner when reaching maximal step-averaged velocity. Specifically, stance-averaged *F*_ver_ increased and the flight time increased, resulting in a longer SL and lower SF. Simultaneously, *F*_ap_ variables and RF decreased (Fig 1). Therefore, for resisted sprints, steps with < 98% of the maximal step-averaged velocity in each trial were used for further analysis. For each variable, the values at every 0.5 m/s in each loading condition were obtained using a cubic spline interpolation technique (Fig 1). In each of loading conditions, the velocity range where data were obtained at every 0.5 m/s from all subjects was 5.0–7.5 m/s for 4 kg, 4.5–7.0 m/s for 6 kg, 4.5–6.5 m/s for 8 kg, 4.0–6.0 m/s for 10 kg, and 4.0–5.5 m/s for 12 kg.

### Statistical analyses

A priori power analysis was performed using GPower (version 3.1.9.7) to calculate the minimum sample size required in repeated-measures analysis of variance (ANOVA) with a desired level of power equal to 0.80, an alpha level set at 0.05, and an effect size (f = 0.4) [25,26]. A required total sample size was 8 subjects. Descriptive data are presented as mean ± SD. Differences between the loading conditions were tested using one-way repeated-measures ANOVA with the Bonferroni post-hoc test. The Greenhouse–Geisser statistic was applied when the sphericity assumption was violated. Eta-squared (*η*^2^) and Cohen’s *d* values were calculated to determine effect size. In addition, the 95% confidence interval for the difference was calculated in post hoc comparisons. Differences in the spatiotemporal and GRF variables at every 0.5 m/s were tested between the conditions in which the data were obtained for all subjects. In all statistical analyses, the significance level was set at *p* < .05. All statistical analyses were performed using SPSS Statistics, version 28 (IBM Corp., Armonk, NY, USA).

## Results

The maximal step-averaged velocity under 0, 4, 6, 8, 10, and 12 kg loading conditions were 9.84 ± 0.41, 8.55 ± 0.41, 8.09 ± 0.33, 7.62 ± 0.34, 7.11 ± 0.31, and 6.71 ± 0.29 m/s, respectively. Compared with the unresisted sprint, the relative velocity losses in the resisted sprints with 4, 6, 8, 10, and 12 kg loads were 13 ± 1, 18 ± 1, 23 ± 2, 28 ± 2, and 32 ± 2%, respectively. Thus, the 4 kg, 6–10 kg, and 12 kg loading conditions were categorized as moderate, heavy, and very heavy resisted sprints, respectively [2].

**Fig 2:**
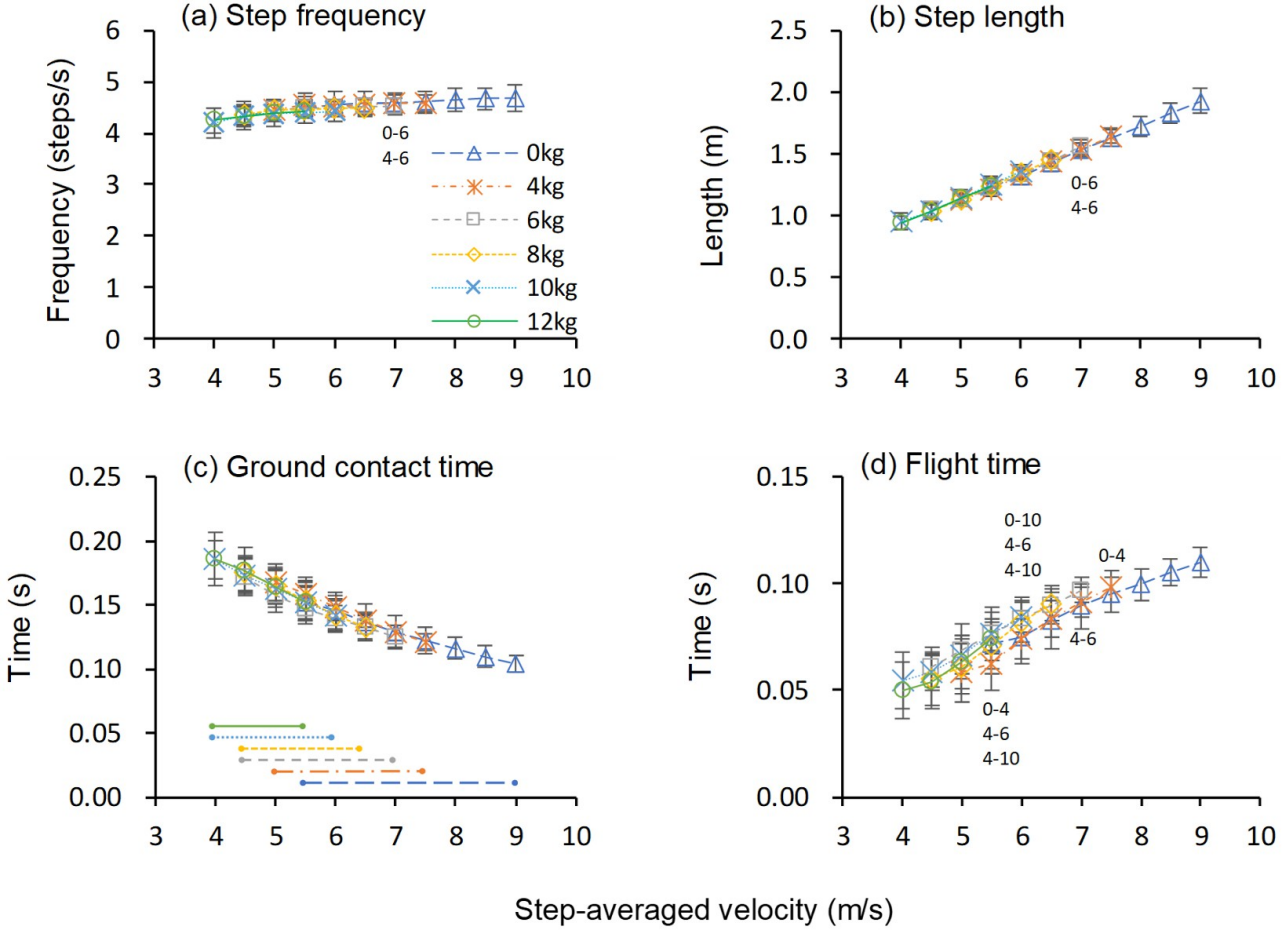
Spatiotemporal variables as a function of step-averaged velocity. Values are means ± standard deviations. Symbols in the figure indicate significant differences between loading conditions (*p* < .05); e.g., “0–10” denotes a significant difference between 0 kg (unresisted sprinting) and 10 kg loading condition. Horizontal lines in panel c indicate velocity range in each loading condition in which values for all subjects were obtained.

Fig 2 shows the changes in spatiotemporal variables as a function of step-averaged velocity.

The one-way repeated measures ANOVA for SF revealed significant main effects of load at 6.0 and 7.0 m/s (6.0 m/s, *F*[4, 32] = 2.820, *p* = .041, *η*^2^ = .261; 7.0 m/s, *F*[2, 16] = 9.00, *p* = .002, *η*^2^ = .529). Significant between-load differences in the SF were found in two cases, where the heavier loading condition was smaller than the lighter one by 0.06 and 0.08 steps/s (1.3 and 1.8%) (Fig 2a, Table 1).

**Table 1:** Combinations of loading conditions with significant differences in spatiotemporal variables.

	Load 1	Load 2	Velocity	Difference (1–2)		95%CI		*p*	Cohen’s *d*
Variable	kg	kg	m/s	%	Absolute	*LL*	*UL*		
Step frequency (steps/s)	0	6	7.0	1.8	0.08	0.01	0.15	.020	0.451
	4	6	7.0	1.3	0.06	0.01	0.10	.012	0.340
Step length (m)	0	6	7.0	-1.8	-0.03	-0.05	0.01	.019	0.451
	4	6	7.0	-1.2	-0.02	-0.03	0.00	.016	0.333
Flight time (s)	0	4	5.5	13.4	0.010	0.004	0.015	< .001	0.720
	4	6	5.5	-19.2	-0.014	-0.026	-0.002	.025	1.103
	4	10	5.5	-22.3	-0.014	-0.025	-0.003	.013	1.344
	0	10	6.0	-11.6	-0.010	-0.018	-0.002	.019	0.939
	4	6	6.0	-11.9	-0.009	-0.017	0.000	.039	0.890
	4	10	6.0	-12.4	-0.010	-0.017	-0.003	.005	1.225
	4	6	7.0	-6.3	-0.006	-0.010	-0.001	.018	0.871
	0	4	7.5	-3.7	-0.004	-0.007	0.000	.038	0.466

This table details the significant differences shown in Fig 2. For example, the top row shows that there was a difference of 1.8% (0.08 step/s) in step frequency between the 0 kg and 6 kg conditions at 7.0 m/s.

The one-way repeated measures ANOVA for SL revealed significant main effects of load at 6.0 and 7.0 m/s (6.0 m/s, *F*[4, 32] = 0.254, *p* = .046, *η*^2^ = .254; 7.0 m/s, *F*[2, 16] = 8.921, *p* = .002, *η*^2^ = .527). Post-hoc comparison showed that in two cases, SL were significantly greater in the heavier condition than in the lighter one by 0.02 and 0.03 m (1.2 and 1.8%) (Fig 2b, Table 1).

The one-way repeated measures ANOVA for ground contact time revealed a significant main effect of load at 5.5 m/s (*F*[5, 40] = 3.026, *p* = .021, *η*^2^ = .274). However, post-hoc comparison showed no significant differences between conditions (Fig 2c).

The one-way repeated measures ANOVA for flight time revealed significant main effects of load from 5.5 to 7.5 m/s (5.5 m/s, *F*[5, 40] = 5.76, *p* < .001, *η*^2^ = .419; 6.0 m/s, *F*[4, 32] = 10.19, *p* < .001, *η*^2^ = .560; 6.5 m/s, *F*[3, 24] = 4.75, *p* = .010, *η*^2^ = .372; 7.0 m/s, *F*[2, 16] = 5.90, *p* = .012, *η*^2^ = .424; 7.5 m/s, *F*[1, 8] = 6.19, *p* = .038, *η*^2^ = .436). Post-hoc comparison showed significant between-load differences in some cases, most of which heavier loading conditions showed longer (0.004–0.014 s or 3.7–22.3%) flight time compared with lighter conditions (Fig 2d, Table 1).

**Fig 3:**
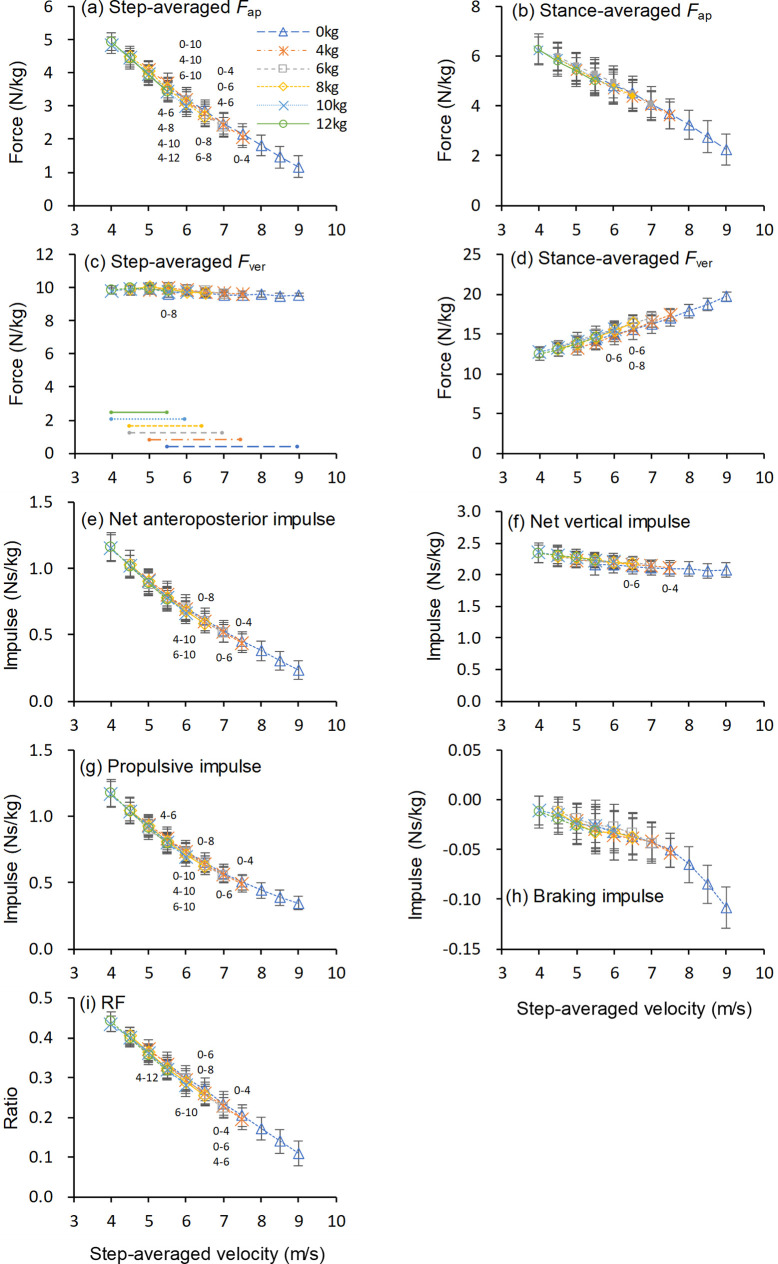
Ground reaction force variables as a function of running velocity. Values are means ± standard deviations. Symbols in the figure indicate significant differences between loading conditions (*p* < .05); e.g., “0–10” denotes a significant difference between 0 kg (unresisted sprint) and 10 kg loading condition. Horizontal lines in panel c indicate velocity range in each loading condition in which values for all subjects were obtained. *F*_ap_: Anteroposterior ground reaction force. *F*_ver_: Vertical ground reaction force. RF: Ratio of *F*_ap_ to resultant ground reaction force.

Fig 3 shows the changes in the GRF variables as functions of the step-averaged velocity.

For step-averaged *F*_ap_, one-way repeated measures ANOVA revealed significant main effects of load from 5.5 to 7.5 m/s (5.5 m/s, *F*[2.32, 18.56] = 6.791, *p* = .005, *η*^2^ = .459; 6.0 m/s, *F*[4, 32] = 11.68, *p* < .001, *η*^2^ = .593; 6.5 m/s, *F*[3, 24] = 7.87, *p* < .001, *η*^2^ = .496; 7.0 m/s, *F*[2, 16] = 18.15, *p* < .001, *η*^2^ = .694; 7.5 m/s, *F*[1, 8] = 14.657, *p* = .005, *η*^2^ = .647). At some running velocities, the lighter condition showed a significantly greater step-averaged *F*_ap_ than the heavier one by 0.03–0.25 N/kg (1.4–7.5%) (Fig 3a, Table 2).

**Table 2:** Combinations of loading conditions with significant differences in ground reaction force variables.

	Load 1	Load 2	Velocity	Difference (1–2)		95%CI		*p*	Cohen’s *d*
Variable	kg	kg	m/s	%	Absolute	*LL*	*UL*		
Step-averaged *F*_ap_ (N/kg)	4	6	5.5	4.3	0.16	0.03	0.28	.014	0.533
	4	8	5.5	4.3	0.16	0.03	0.29	.017	0.509
	4	10	5.5	6.2	0.23	0.11	0.34	< .001	0.760
	4	12	5.5	6.8	0.25	0.01	0.48	.036	0.814
	0	10	6.0	7.5	0.24	0.07	0.41	.006	0.742
	4	10	6.0	6.3	0.20	0.07	0.33	.004	0.591
	6	10	6.0	5.6	0.18	0.05	0.30	.006	0.560
	0	8	6.5	6.2	0.18	0.03	0.33	.019	0.544
	6	8	6.5	2.1	0.06	0.02	0.10	.009	0.180
	0	4	7.0	1.4	0.03	0.00	0.07	.031	0.104
	0	6	7.0	4.4	0.11	0.05	0.17	.002	0.344
	4	6	7.0	3.0	0.07	0.01	0.14	.032	0.236
	0	4	7.5	4.2	0.09	0.04	0.14	.005	0.280
Step-averaged *F*_ver_ (N/kg)	0	8	5.5	-3.1	-0.30	-0.49	-0.11	.002	1.546
Stance-averaged *F*_ver_ (N/kg)	0	6	6.0	-4.7	0.70	-1.39	-0.01	.047	0.612
	0	6	6.5	-5.2	-0.81	-1.54	-0.09	.027	0.743
	0	8	6.5	-4.8	-0.74	-1.44	-0.05	.035	0.643
Net anteroposterior impulse (Ns/kg)	4	6	6.0	5.2	0.04	0.00	0.06	.023	0.364
	6	10	6.0	4.1	0.03	0.00	0.06	.039	0.349
	0	8	6.5	4.7	0.03	0.00	0.06	.047	0.366
	0	6	7.0	2.9	0.02	0.00	0.03	.039	0.206
	0	4	7.5	3.5	0.02	0.00	0.03	.015	0.221
Propulsive impulse (Ns/kg)	4	6	5.5	1.9	0.02	0.01	0.06	.016	0.467
	0	10	6.0	5.2	0.04	0.00	0.08	.045	0.503
	4	10	6.0	4.8	0.04	0.01	0.06	.004	0.502
	6	10	6.0	3.4	0.02	0.01	0.04	.013	0.349
	0	8	6.5	4.5	0.03	0.00	0.06	.036	0.438
	0	6	7.0	2.6	0.01	0.00	0.03	.039	0.235
	0	4	7.5	2.6	0.01	0.00	0.02	.012	0.219
Net vertical Impulse (Ns/kg)	0	6	6.5	-2.8	-0.06	-0.10	-0.02	.006	0.343
	0	4	7.5	-1.3	-0.03	-0.04	-0.01	.002	0.251
RF	4	12	5.0	4.2	0.016	0.002	0.029	.019	0.668
	6	10	6.0	4.9	0.014	0.006	0.023	.002	0.528
	0	6	6.5	4.8	0.013	0.001	0.025	.026	0.476
	0	8	6.5	5.9	0.016	0.002	0.029	.023	0.583
	0	4	7.0	2.1	0.005	0.000	0.010	.042	0.172
	0	6	7.0	2.3	0.010	0.004	0.017	.005	0.375
	4	6	7.0	2.4	0.005	0.001	0.010	.021	0.199
	0	4	7.5	4.3	0.009	0.004	0.014	.003	0.323

This table details the significant differences shown in Fig 3. For example, the top row shows that there was a difference of 4.3% (0.16 N/kg) in step-averaged *F*_ap_ between the 4 kg and 6 kg conditions at 5.5 m/s. *F*_ap_, anteroposterior ground reaction force; *F*_ver_, vertical ground reaction force; RF, ratio of stance-averaged *F*_ap_ to resultant force.

There were no significant main effects of load for the stance-averaged *F*_ap_ (Fig 3b).

The one-way repeated measures ANOVA for step-averaged *F*_ver_ revealed significant main effect of load at 5.5 (*F*[2.83, 22.61] = 5.233, *p* = .008, *η*^2^ = .395). A post-hoc comparison showed that the step-averaged *F*_ver_ at 5.5 m/s in the 8 kg condition was significantly greater than that in unresisted sprint by 0.30 N/kg (3.1%) (Fig 3c, Table 2).

The one-way repeated measures ANOVA for stance-averaged *F*_ver_ revealed significant main effects of load from 5.0 to 7.0 m/s (5.0 m/s, *F*[4, 32] = 3.68, *p* = .014, *η*^2^ = .315; 5.5 m/s, *F*[5, 40] = 4.63, *p* = .002, *η*^2^ = .366; 6.0 m/s, *F*[4, 32] = 6.14, *p* < .001, *η*^2^ = .434; 6.5 m/s, *F*[3, 24] = 6.73, *p* = .002, *η*^2^ = .457; 7.0 m/s, *F*[2, 16] = 5.18, *p* = .018, *η*^2^ = .393). Post-hoc comparison showed that stance-averaged *F*_ver_ was significantly greater in the heavier condition than in the lighter condition by 0.70–0.81 N/kg (4.7–5.2%) in three cases (Fig 3d, Table 2).

The one-way repeated measures ANOVA for net anteroposterior impulse revealed significant main effects of load from 6.0 to 7.5 m/s (6.0 m/s, *F*[4, 32] = 5.82, *p* = .001, *η*^2^ = .421; 6.5 m/s, *F*[3, 24] = 4.44, *p* = .013, *η*^2^ = .357; 7.0 m/s, *F*[2, 16] = 5.04, *p* = .002, *η*^2^ = .387; 7.5 m/s, *F*[1, 8] = 9.567, *p* = .015, *η*^2^ = .545). Post-hoc comparison showed that net anteroposterior impulse was significantly greater in the lighter condition than in the heavier condition by 0.02–0.04 Ns/kg (2.9–5.2%) in five cases (Fig 3e, Table 2).

The one-way repeated measures ANOVA for propulsive impulse revealed significant main effects of load from 5.5 to 7.5 m/s (5.5 m/s, *F*[5, 40] = 2.47, *p* = .049, *η*^2^ = .236; 6.0 m/s, *F*[4, 32] = 7.68, *p* < .001, *η*^2^ = .490; 6.5 m/s, *F*[3, 24] = 5.21, *p* = .006, *η*^2^ = .395; 7.0 m/s, *F*[2, 16] = 6.18, *p* = .001, *η*^2^ = .436; 7.5 m/s, *F*[1, 8] = 10.56, *p* = .012, *η*^2^ = .569). Significant between-load differences in the propulsive impulse were found in some cases, where the lighter loading condition was greater than the heavier one by 0.01–0.04 Ns/kg (1.9–5.2%) (Fig 3g, Table 2).

There were no significant main effects of load for braking impulse (Fig 3h).

The one-way repeated measures ANOVA for net vertical impulse revealed significant main effects of load from 6.5 to 7.5 m/s (6.5 m/s, *F*[3, 24] = 3.92, *p* = .021, *η*^2^ = .329; 7.0 m/s, *F*[1.25, 9.99] = 4.70, *p* = .049, *η*^2^ = .370; 7.5 m/s, *F*[1, 8] = 20.07, *p* = .002, *η*^2^ = .715). Post-hoc comparison showed that net vertical impulse was significantly greater in the heavier condition by 0.03 and 0.06 Ns/kg (1.3 and 2.8%) in two cases (Fig 3f, Table 2).

The one-way repeated measures ANOVA for RF revealed significant main effects of load from 5.0 to 7.5 m/s (5.0 m/s, *F*[4, 32] = 5.34, *p* = .002, *η*^2^ = .400; 5.5 m/s, *F*[5, 40] = 4.40, *p* = .003, *η*^2^ = .355; 6.0 m/s, *F*[4, 32] = 6.49, *p* < .001, *η*^2^ = .448; 6.5 m/s, *F*[3, 24] = 10.97, *p* < .001, *η*^2^ = .578; 7.0 m/s, *F*[2, 16] = 16.63, *p* < .001, *η*^2^ = .675; 7.5 m/s, *F*[1, 8] = 17.94, *p* = .003, *η*^2^ = .692). Post-hoc comparison showed that RF was significantly greater in the lighter condition than in the heavier condition by 0.005–0.016 (2.1–5.9%) in some cases (Fig 3i, Table 2).

## Discussion

In contrast to our hypothesis, the ANOVAs showed significant effects of load on most of the analyzed spatiotemporal and GRF variables. These results indicate that the horizontal loading influences the sprint mechanics, even when kinetic and kinematic variables are compared at a given running velocity. However, it should be noted that most of the significant inter-load differences were observed for the values at the maximum velocity under each loading condition. The observed differences were at most small percentage (approximately 4% for the GRF and 1.5% for spatiotemporal variables) except for flight time (3.7–22.3%) (Figs 2 and 3 and Tables 1 and 2). Furthermore, significant differences between the heaviest (12 kg) loading condition and the other loading conditions were found in only two cases in the GRF variable (Figs 2 and 3, Tables 1 and 2). These results indicate that, at least until the point at which the maximum velocity appeared in each loading condition, the spatiotemporal and GRF variables during resisted sprints are similar to those in the corresponding velocity stage during an unresisted sprint, even under heavy loading conditions. In other words, resisted sprints with different loads can be a method “to train the force production capacity at the specific targeted velocity for an extended time, to maximize adaptations” [18,27]. Notably, the current results indicate that, in contrast to the recommendation [1,4], resisted sprints with heavy load can be a training modality, being specific to improve the sprinting ability in the initial stage of maximal sprint acceleration. For example, based on the results of this study, a 10 or 12 kg load would be appropriate for training the first few steps, a 6 or 8 kg load for the 0–5 m section, which corresponds to running velocity of 0–6.5 m/s, and a 4 kg load for the 5–10 m section, which corresponds to 6.5–8.0 m/s. In the later section, a load of less than 4 kg would be appropriate. However, what is important is not the absolute load, but the degree of velocity loss caused by the load. The relationship between absolute load and velocity loss will differ among individuals, notably in relation with the strength level of the practitioners. Thus, it is necessary to examine the individual’s absolute load-running velocity relationship when determining the load used in the resisted sprint training.

In contrast to the present results, previous studies have reported considerable differences in sprint mechanics between unresisted and heavy resisted sprints [4,6]. The discrepancy occurred possibly because previous studies compared values at a given distance or step number, while the present study at a given velocity. At a given distance or step number, the heavier the resistance load, the slower the running velocity. Previous studies reported that the running velocity at a given distance (5 m) or step number (second step) in resisted sled sprints with a load of approximately 30% body mass decreased by around 23% (unresisted vs resisted; 5.7 m/s vs 4.4 m/s [4] or 4.2 m/s vs 3.3 m/s [6]). With this difference in running velocity, they found a 14–20% increase in ground contact time, 40–50% decrease in flight time, 24% decrease in SL, 43% decrease in average effective *F*_ver_ during ground contact, 29% increase in net horizontal impulse, and 39% increase in the mean ratio of forces applied to the ground in heavy resisted sprint [4,6]. These values are similar to the differences between 5.5 and 4.5 m/s for the corresponding variables in this study. The present results showed that ground contact time was ~15% longer, flight time was ~23% shorter, SL was ~16% smaller, stance-averaged (effective) *F*_ver_ was ~31% lower, net anteroposterior impulse was ~32% larger, and RF was ~25% larger at 4.5 m/s than at 5.5 m/s (Figs 2 and 3). Therefore, the differences in spatiotemporal and GRF variables between unresisted and resisted conditions in previous studies could be mainly attributed to the difference in running velocity due to that in the magnitude of the resisted loads.

However, the present results also indicate that sprint mechanics near the maximum velocity in resisted sprints could be slightly different from corresponding velocity in unresisted sprint, regardless of the load magnitude. Notably, the observed changes in the spatiotemporal and GRF variables with horizontal loading are in contrast to those reported in previous studies [1,3–6], which observed that resistance loads caused a longer contact time and a more horizontal direction of force and impulse application to the ground. In contrast, the present results showed that the resisted sprints changed the force applied to the ground to be more vertical and yielded a longer flight time (Figs 2 and 3, Tables 1 and 2). While reasons for the more vertical orientation of the GRF cannot be clarified from the data obtained in this study, there is a possibility that the subjects might have strained and exerted force upward while trying to maintain velocity against the resistive load. Nevertheless, this result indicates the possibility that training using resisted sprints with an excessively long distance or duration would cause changes in movement pattern into a more vertical force application to the ground, which is detrimental to sprint performance [23,24,28]. A previous study reported that 8-week resisted sprint training program improved sprint acceleration performance with a decrease in vertical impulse [29]. Therefore, the phenomenon of more vertical force application near the maximal velocity in resisted sprint may not have adverse impacts on kinetic adaptations through resisted sprint training.

In the present study, the number of trials for each loading condition was one, because of the time available at the experimental facility and to prevent the effects of fatigue. Therefore, the results of this study would include intra-individual variation. However, it is known that the reproducibility of spatiotemporal and GRF variables in resisted and unresisted sprint is very high in athletes [7,13,30]. We limited the subjects to sprinters who have experienced resisted sprinting in their own training programs as a routine. In addition, the spatiotemporal and GRF values obtained in previous studies were similar to those reported previous studies [4,6,10]. Thus, we believe that the main findings of the present study were not affected by the insufficient number of trials. On the other hand, we adopted steps at less than 98% of the maximal step-averaged velocity in each trial for resisted sprints because some subjects abruptly changed their sprinting mechanics when reaching maximal velocity in each resisted sprint (Fig 1). The causes of this phenomenon and its impact on training efficacy are unknown. However, it is of significant interest to develop effective resisted sprint training, including optimal distance and instructions for athletes. Further studies are required to elucidate the physiological mechanism of this phenomenon.

Finally, we would like to note some limitations and prospects. The present study lacks analysis aiming to clarify the influences of horizontal resistance loads on the runner’s movements, i.e., kinetics and kinematics of body segments and joints. Further studies including high-speed video analysis are required to examine this. In addition, the subjects in this study were limited to well-trained male college sprinters. It is possible that the influence of horizontal resistance load on sprinting behavior differs depending on gender, age, running technique or physical fitness level. These aspects will also be topics for future research.

## Conclusions

We investigated the influence of horizontal resistance load on the spatiotemporal and GRF variables at a given running velocity during maximal sprinting. The results showed that the differences between loading conditions in spatiotemporal and GRF variables at a given running velocity were much smaller than, or even opposite to, those reported in previous studies that performed comparisons between loading conditions at a given distance or step number. This indicates that nature of resisted sprints can simulate various stages of unresisted maximal sprint acceleration by changing the magnitude of the resisted load. Therefore, selecting a heavy load in resisted sprint training could be a modality for improving sprint performance in the low-velocity range. This idea is supported by recent reports indicating the effectiveness of resisted sprint training with heavy load. However, the fact that sprint mechanics changed in resisted sprints near the maximal velocity under each loading condition suggests that when implementing resisted sprint training, athletes and their coaches need to be careful in setting the optimal running distance in accordance with the magnitude of the resisted loads.

## Supporting information

S1 File Individual values of spatiotemporal and GRF variables. (PDF)
