# Supplementary material for: Influence of horizontal resistance loads on spatiotemporal and ground reaction force variables during maximal sprint acceleration
Source: PLoS One. 2023 Dec 12;18(12):e0295758. doi: 10.1371/journal.pone.0295758 (PMC10715639; doi:10.1371/journal.pone.0295758)
Supplement: S1 File — (PDF) [file pone.0295758.s001.pdf]

Individual values of step frequency (steps/s)

		4.0 m/s		4.5 m/s				5.0 m/s					5.5 m/s					
		Load (kg)		Load (kg)				Load (kg)					Load (kg)					
	Specialty	10	12	6	8	10	12	4	6	8	10	12	0	4	6	8	10	12
Sub. A	100 m	4.40	4.34	4.54	4.50	4.48	4.47	4.62	4.58	4.54	4.43	4.50	4.63	4.63	4.58	4.53	4.38	4.36
Sub. B	100 m	4.51	4.64	4.41	4.61	4.68	4.60	4.75	4.64	4.69	4.74	4.55	4.84	4.78	4.72	4.68	4.61	4.59
Sub. C	200m, 400 m	4.48	4.42	4.43	4.50	4.60	4.58	4.52	4.47	4.64	4.57	4.65	4.63	4.63	4.56	4.68	4.67	4.66
Sub. D	100 m	3.85	3.92	4.05	4.13	3.96	4.12	4.28	4.28	4.30	3.99	4.25	4.11	4.38	4.39	4.40	4.07	4.34
Sub.E	100 m	3.72	3.90	3.99	3.99	3.91	4.05	4.17	4.14	4.06	4.10	4.20	4.27	4.34	4.23	4.11	4.19	4.22
Sub. F	100 m	4.17	4.19	4.30	4.31	4.29	4.24	4.37	4.47	4.36	4.33	4.34	4.43	4.52	4.46	4.37	4.34	4.35
Sub. G	100 m	3.98	4.22	4.20	4.25	4.16	4.18	4.24	4.25	4.34	4.24	4.23	4.06	4.34	4.30	4.43	4.24	4.30
Sub. H	100 m	4.34	4.30	4.46	4.51	4.55	4.46	4.62	4.64	4.56	4.55	4.49	4.73	4.70	4.62	4.60	4.58	4.52
Sub. I	100 m	4.29	4.29	4.52	4.43	4.47	4.34	4.37	4.53	4.44	4.53	4.41	4.69	4.48	4.50	4.40	4.55	4.53

		6.0 m/s					6.5 m/s				7.0 m/s			7.5 m/s		8.0 m/s	8.5 m/s	9.0 m/s
		Load (kg)					Load (kg)				Load (kg)			Load (kg)		Load (kg)	Load (kg)	Load (kg)
	Specialty	0	4	6	8	10	0	4	6	8	0	4	6	0	4	0	0	0
Sub. A	100 m	4.62	4.56	4.61	4.46	4.39	4.64	4.59	4.52	4.43	4.60	4.57	4.48	4.57	4.52	4.59	4.59	4.53
Sub. B	100 m	4.86	4.61	4.66	4.67	4.66	4.86	4.63	4.69	4.69	4.82	4.82	4.72	4.85	4.87	4.98	4.98	5.01
Sub. C	200m, 400 m	4.68	4.61	4.57	4.67	4.69	4.69	4.69	4.63	4.70	4.73	4.73	4.69	4.77	4.71	4.81	4.83	4.86
Sub. D	100 m	4.23	4.47	4.43	4.46	4.15	4.32	4.49	4.42	4.45	4.41	4.50	4.41	4.44	4.51	4.48	4.51	4.53
Sub.E	100 m	4.34	4.29	4.22	4.19	4.21	4.37	4.34	4.25	4.15	4.41	4.34	4.24	4.35	4.29	4.39	4.41	4.39
Sub. F	100 m	4.52	4.51	4.47	4.39	4.36	4.51	4.42	4.48	4.40	4.46	4.44	4.45	4.50	4.48	4.50	4.52	4.51
Sub. G	100 m	4.20	4.35	4.34	4.37	4.32	4.26	4.33	4.35	4.41	4.34	4.38	4.34	4.41	4.41	4.41	4.42	4.42
Sub. H	100 m	4.82	4.73	4.62	4.61	4.52	4.81	4.76	4.62	4.59	4.81	4.72	4.65	4.79	4.69	4.82	4.83	4.85
Sub. I	100 m	4.72	4.51	4.55	4.52	4.52	4.72	4.58	4.62	4.58	4.75	4.63	4.63	4.80	4.65	4.82	4.87	5.00

Individual values of step length (m)

		4.0 m/s		4.5 m/s				5.0 m/s					5.5 m/s					
		Load (kg)		Load (kg)				Load (kg)					Load (kg)					
	Specialty	10	12	6	8	10	12	4	6	8	10	12	0	4	6	8	10	12
Sub. A	100 m	0.91	0.92	0.99	1.00	1.00	1.01	1.08	1.09	1.10	1.13	1.11	1.19	1.19	1.20	1.22	1.26	1.26
Sub. B	100 m	0.88	0.86	1.02	0.97	0.96	0.98	1.05	1.08	1.07	1.05	1.10	1.14	1.15	1.17	1.17	1.19	1.20
Sub. C	200m, 400 m	0.89	0.90	1.01	1.00	0.98	0.98	1.11	1.12	1.08	1.09	1.08	1.19	1.19	1.21	1.18	1.18	1.18
Sub. D	100 m	1.04	1.02	1.11	1.09	1.14	1.09	1.17	1.17	1.16	1.25	1.18	1.34	1.26	1.25	1.25	1.35	1.27
Sub.E	100 m	1.07	1.03	1.12	1.13	1.15	1.11	1.20	1.21	1.23	1.22	1.19	1.29	1.27	1.30	1.34	1.31	1.30
Sub. F	100 m	0.96	0.95	1.04	1.04	1.05	1.06	1.14	1.12	1.15	1.16	1.15	1.24	1.21	1.24	1.26	1.27	1.26
Sub. G	100 m	1.01	0.95	1.07	1.06	1.08	1.08	1.18	1.18	1.15	1.18	1.18	1.35	1.27	1.28	1.24	1.30	1.28
Sub. H	100 m	0.92	0.93	1.01	0.99	0.99	1.01	1.08	1.08	1.10	1.10	1.11	1.16	1.17	1.19	1.19	1.20	1.22
Sub. I	100 m	0.93	0.94	1.00	1.02	1.01	1.04	1.14	1.11	1.13	1.11	1.13	1.18	1.23	1.22	1.25	1.21	1.21

		6.0 m/s					6.5 m/s				7.0 m/s			7.5 m/s		8.0 m/s	8.5 m/s	9.0 m/s
		Load (kg)					Load (kg)				Load (kg)			Load (kg)		Load (kg)	Load (kg)	Load (kg)
	Specialty	0	4	6	8	10	0	4	6	8	0	4	6	0	4	0	0	0
Sub. A	100 m	1.30	1.32	1.30	1.35	1.37	1.40	1.42	1.44	1.47	1.52	1.53	1.56	1.64	1.66	1.74	1.85	1.99
Sub. B	100 m	1.23	1.30	1.29	1.29	1.29	1.34	1.40	1.39	1.39	1.45	1.45	1.48	1.54	1.54	1.60	1.71	1.80
Sub. C	200m, 400 m	1.28	1.30	1.31	1.28	1.28	1.39	1.39	1.41	1.38	1.48	1.48	1.49	1.57	1.59	1.66	1.76	1.85
Sub. D	100 m	1.42	1.34	1.35	1.34	1.44	1.50	1.45	1.47	1.46	1.59	1.56	1.59	1.69	1.66	1.79	1.89	1.99
Sub.E	100 m	1.38	1.40	1.42	1.43	1.43	1.49	1.50	1.53	1.57	1.59	1.62	1.65	1.72	1.75	1.82	1.93	2.05
Sub. F	100 m	1.33	1.34	1.34	1.37	1.38	1.44	1.47	1.46	1.48	1.57	1.58	1.57	1.67	1.68	1.78	1.88	2.00
Sub. G	100 m	1.43	1.38	1.39	1.38	1.39	1.53	1.50	1.50	1.48	1.61	1.60	1.62	1.70	1.70	1.81	1.92	2.04
Sub. H	100 m	1.25	1.27	1.30	1.30	1.33	1.35	1.37	1.41	1.42	1.46	1.48	1.51	1.57	1.60	1.66	1.76	1.86
Sub. I	100 m	1.27	1.33	1.32	1.33	1.33	1.38	1.42	1.41	1.42	1.48	1.52	1.51	1.56	1.62	1.66	1.75	1.80

Individual values of ground contact time (s)

		4.0 m/s		4.5 m/s				5.0 m/s					5.5 m/s					
		Load (kg)		Load (kg)				Load (kg)					Load (kg)					
	Specialty	10	12	6	8	10	12	4	6	8	10	12	0	4	6	8	10	12
Sub. A	100 m	0.186	0.183	0.161	0.186	0.174	0.177	0.170	0.149	0.183	0.167	0.166	0.163	0.164	0.151	0.169	0.160	0.157
Sub. B	100 m	0.160	0.148	0.164	0.161	0.158	0.152	0.149	0.163	0.151	0.154	0.149	0.136	0.147	0.148	0.138	0.138	0.144
Sub. C	200m, 400 m	0.173	0.191	0.157	0.166	0.154	0.176	0.159	0.152	0.154	0.157	0.164	0.141	0.148	0.150	0.142	0.140	0.142
Sub. D	100 m	0.201	0.198	0.194	0.182	0.193	0.193	0.162	0.167	0.161	0.180	0.192	0.165	0.162	0.149	0.153	0.166	0.165
Sub.E	100 m	0.202	0.217	0.188	0.185	0.191	0.199	0.185	0.170	0.184	0.186	0.171	0.158	0.168	0.156	0.179	0.170	0.162
Sub. F	100 m	0.193	0.205	0.187	0.193	0.188	0.202	0.195	0.175	0.171	0.170	0.176	0.178	0.186	0.159	0.162	0.163	0.168
Sub. G	100 m	0.173	0.185	0.161	0.177	0.165	0.169	0.161	0.148	0.168	0.155	0.153	0.159	0.154	0.138	0.155	0.149	0.151
Sub. H	100 m	0.183	0.185	0.171	0.173	0.168	0.161	0.170	0.156	0.169	0.150	0.150	0.144	0.153	0.147	0.146	0.145	0.143
Sub. I	100 m	0.197	0.163	0.161	0.150	0.165	0.166	0.159	0.136	0.142	0.142	0.158	0.125	0.147	0.126	0.135	0.134	0.139

		6.0 m/s					6.5 m/s				7.0 m/s			7.5 m/s		8.0 m/s	8.5 m/s	9.0 m/s
		Load (kg)					Load (kg)				Load (kg)			Load (kg)		Load (kg)	Load (kg)	Load (kg)
	Specialty	0	4	6	8	10	0	4	6	8	0	4	6	0	4	0	0	0
Sub. A	100 m	0.156	0.159	0.146	0.154	0.154	0.149	0.147	0.138	0.148	0.139	0.132	0.126	0.127	0.126	0.122	0.118	0.106
Sub. B	100 m	0.138	0.136	0.136	0.131	0.130	0.133	0.131	0.127	0.124	0.124	0.122	0.118	0.119	0.116	0.111	0.103	0.100
Sub. C	200m, 400 m	0.136	0.143	0.139	0.136	0.129	0.126	0.138	0.126	0.124	0.117	0.120	0.120	0.112	0.115	0.108	0.103	0.099
Sub. D	100 m	0.157	0.157	0.151	0.149	0.156	0.153	0.141	0.146	0.141	0.148	0.129	0.135	0.139	0.126	0.129	0.120	0.112
Sub.E	100 m	0.151	0.150	0.146	0.159	0.151	0.135	0.139	0.137	0.142	0.127	0.131	0.137	0.124	0.122	0.118	0.112	0.107
Sub. F	100 m	0.168	0.162	0.148	0.153	0.155	0.158	0.139	0.141	0.142	0.146	0.138	0.130	0.137	0.129	0.126	0.113	0.111
Sub. G	100 m	0.150	0.145	0.134	0.136	0.142	0.134	0.133	0.131	0.128	0.126	0.127	0.125	0.121	0.125	0.117	0.114	0.109
Sub. H	100 m	0.134	0.136	0.137	0.133	0.136	0.127	0.136	0.133	0.128	0.122	0.133	0.127	0.116	0.121	0.112	0.107	0.103
Sub. I	100 m	0.120	0.142	0.120	0.123	0.125	0.114	0.133	0.115	0.116	0.110	0.122	0.111	0.107	0.108	0.101	0.094	0.092

Individual values of flight time (s)

		4.0 m/s		4.5 m/s				5.0 m/s					5.5 m/s					
		Load (kg)		Load (kg)				Load (kg)					Load (kg)					
	Specialty	10	12	6	8	10	12	4	6	8	10	12	0	4	6	8	10	12
Sub. A	100 m	0.041	0.047	0.059	0.037	0.050	0.048	0.047	0.070	0.038	0.059	0.057	0.053	0.052	0.067	0.052	0.069	0.072
Sub. B	100 m	0.062	0.067	0.063	0.056	0.056	0.065	0.061	0.052	0.063	0.057	0.071	0.071	0.062	0.064	0.075	0.079	0.074
Sub. C	200m, 400 m	0.051	0.035	0.068	0.056	0.064	0.042	0.062	0.072	0.062	0.062	0.051	0.076	0.068	0.069	0.072	0.074	0.073
Sub. D	100 m	0.059	0.057	0.054	0.060	0.060	0.049	0.071	0.066	0.072	0.071	0.044	0.079	0.066	0.079	0.074	0.080	0.066
Sub.E	100 m	0.067	0.040	0.062	0.065	0.066	0.049	0.056	0.072	0.062	0.059	0.067	0.076	0.063	0.080	0.064	0.069	0.075
Sub. F	100 m	0.046	0.034	0.046	0.039	0.046	0.034	0.033	0.049	0.059	0.061	0.055	0.048	0.036	0.065	0.067	0.068	0.062
Sub. G	100 m	0.079	0.052	0.078	0.058	0.076	0.070	0.075	0.088	0.062	0.081	0.083	0.089	0.077	0.095	0.071	0.086	0.082
Sub. H	100 m	0.048	0.048	0.054	0.049	0.052	0.063	0.047	0.060	0.051	0.070	0.074	0.068	0.060	0.069	0.072	0.074	0.079
Sub. I	100 m	0.038	0.070	0.061	0.075	0.060	0.065	0.070	0.085	0.083	0.080	0.069	0.089	0.077	0.096	0.092	0.087	0.082

		6.0 m/s					6.5 m/s				7.0 m/s			7.5 m/s		8.0 m/s	8.5 m/s	9.0 m/s
		Load (kg)					Load (kg)				Load (kg)			Load (kg)		Load (kg)	Load (kg)	Load (kg)
	Specialty	0	4	6	8	10	0	4	6	8	0	4	6	0	4	0	0	0
Sub. A	100 m	0.060	0.060	0.071	0.070	0.074	0.066	0.070	0.084	0.077	0.079	0.086	0.097	0.092	0.095	0.096	0.099	0.114
Sub. B	100 m	0.068	0.081	0.078	0.083	0.084	0.073	0.085	0.086	0.089	0.083	0.086	0.094	0.087	0.089	0.089	0.098	0.099
Sub. C	200m, 400 m	0.078	0.075	0.080	0.078	0.084	0.087	0.076	0.090	0.088	0.094	0.092	0.094	0.097	0.097	0.100	0.104	0.106
Sub. D	100 m	0.079	0.067	0.075	0.075	0.084	0.078	0.082	0.081	0.083	0.079	0.093	0.092	0.087	0.095	0.094	0.102	0.109
Sub.E	100 m	0.080	0.084	0.091	0.080	0.087	0.094	0.091	0.098	0.099	0.099	0.099	0.099	0.106	0.111	0.110	0.115	0.121
Sub. F	100 m	0.054	0.061	0.076	0.075	0.074	0.064	0.088	0.083	0.086	0.078	0.088	0.095	0.085	0.095	0.096	0.108	0.111
Sub. G	100 m	0.088	0.085	0.097	0.094	0.090	0.101	0.098	0.099	0.099	0.105	0.102	0.106	0.105	0.102	0.109	0.112	0.117
Sub. H	100 m	0.074	0.076	0.079	0.084	0.085	0.081	0.075	0.084	0.090	0.087	0.079	0.089	0.092	0.092	0.096	0.100	0.104
Sub. I	100 m	0.093	0.081	0.100	0.098	0.097	0.098	0.085	0.102	0.103	0.101	0.094	0.105	0.102	0.108	0.107	0.111	0.109

Individual values of step-averaged anteroposterior ground reaction force (N/kg)

		4.0 m/s		4.5 m/s				5.0 m/s					5.5 m/s					
		Load (kg)		Load (kg)				Load (kg)					Load (kg)					
	Specialty	10	12	6	8	10	12	4	6	8	10	12	0	4	6	8	10	12
Sub. A	100 m	4.42	4.48	3.91	4.17	4.11	4.04	3.74	3.43	3.80	3.48	3.42	3.08	3.18	3.12	3.10	2.96	2.79
Sub. B	100 m	4.97	4.81	4.47	4.49	4.75	4.45	4.02	3.95	4.01	4.18	3.94	3.27	3.54	3.30	3.54	3.42	3.61
Sub. C	200m, 400 m	4.71	4.77	4.14	4.26	4.37	4.43	3.92	3.73	3.90	4.04	3.95	3.46	3.64	3.48	3.41	3.26	3.24
Sub. D	100 m	5.09	5.17	4.80	4.87	4.58	4.82	4.53	4.32	4.39	4.23	4.46	3.98	4.19	3.97	4.02	3.87	3.72
Sub.E	100 m	4.54	4.82	4.19	4.25	4.18	4.05	3.70	3.83	3.77	3.94	3.63	3.32	3.40	3.42	3.17	3.23	3.29
Sub. F	100 m	4.73	4.94	4.40	4.29	4.38	4.46	3.92	3.96	3.81	3.79	3.88	3.59	3.61	3.49	3.42	3.42	3.34
Sub. G	100 m	4.87	5.37	4.85	5.11	4.57	4.60	4.45	4.25	4.62	4.31	4.17	3.89	4.03	3.89	3.97	3.82	3.76
Sub. H	100 m	5.07	5.13	4.62	4.59	4.46	4.37	4.37	4.09	4.15	3.93	3.83	3.95	3.84	3.61	3.53	3.57	3.55
Sub. I	100 m	5.03	4.87	4.55	4.44	4.58	4.60	3.97	3.84	3.93	3.85	4.11	3.43	3.59	3.34	3.43	3.42	3.49

		6.0 m/s					6.5 m/s				7.0 m/s			7.5 m/s		8.0 m/s	8.5 m/s	9.0 m/s
		Load (kg)					Load (kg)				Load (kg)			Load (kg)		Load (kg)	Load (kg)	Load (kg)
	Specialty	0	4	6	8	10	0	4	6	8	0	4	6	0	4	0	0	0
Sub. A	100 m	2.59	2.60	2.73	2.54	2.43	2.29	2.28	2.30	2.22	1.93	1.89	1.91	1.70	1.55	1.31	0.94	0.58
Sub. B	100 m	3.11	2.97	3.03	3.04	2.98	2.76	2.67	2.59	2.60	2.30	2.27	2.17	2.00	1.93	1.63	1.27	0.95
Sub. C	200m, 400 m	3.10	3.18	3.13	2.98	2.90	2.68	2.77	2.63	2.55	2.36	2.34	2.34	2.02	1.95	1.67	1.28	1.03
Sub. D	100 m	3.67	3.75	3.73	3.75	3.43	3.27	3.21	3.26	3.21	2.83	2.76	2.76	2.50	2.44	2.09	1.68	1.41
Sub.E	100 m	3.00	2.83	2.85	2.73	2.69	2.56	2.42	2.44	2.43	2.18	2.14	2.06	1.81	1.78	1.60	1.27	1.01
Sub. F	100 m	3.34	3.08	3.07	3.09	2.88	2.94	2.58	2.63	2.54	2.36	2.36	2.26	1.96	1.95	1.71	1.34	1.13
Sub. G	100 m	3.54	3.57	3.60	3.35	3.36	3.17	3.09	3.20	3.10	2.88	2.82	2.69	2.58	2.42	2.19	1.86	1.51
Sub. H	100 m	3.57	3.47	3.26	3.20	3.15	3.25	3.16	2.95	2.87	2.83	2.86	2.68	2.47	2.45	2.22	1.95	1.66
Sub. I	100 m	3.11	3.22	3.07	3.05	3.03	2.81	2.84	2.67	2.62	2.52	2.46	2.36	2.22	2.01	1.81	1.55	1.20

Individual values of stance-averaged anteroposterior ground reaction force (N/kg)

		4.0 m/s		4.5 m/s				5.0 m/s					5.5 m/s					
		Load (kg)		Load (kg)				Load (kg)					Load (kg)					
	Specialty	10	12	6	8	10	12	4	6	8	10	12	0	4	6	8	10	12
Sub. A	100 m	5.37	5.58	5.30	4.95	5.22	5.07	4.73	4.96	4.54	4.62	4.50	4.00	4.11	4.43	3.97	4.15	4.02
Sub. B	100 m	6.81	6.89	6.15	5.97	6.36	6.26	5.59	5.15	5.59	5.65	5.71	4.89	4.94	4.66	5.38	5.27	5.36
Sub. C	200m, 400 m	6.08	5.62	5.87	5.64	6.10	5.44	5.40	5.40	5.38	5.57	5.13	5.23	5.23	5.02	5.04	4.90	4.81
Sub. D	100 m	6.56	6.59	6.18	6.45	5.93	6.00	6.45	6.06	6.28	5.83	5.42	5.78	5.83	5.99	5.89	5.64	5.12
Sub.E	100 m	5.97	5.66	5.55	5.69	5.56	5.00	4.82	5.43	5.00	5.12	4.98	4.86	4.61	5.10	4.27	4.46	4.72
Sub. F	100 m	5.82	5.73	5.45	5.12	5.40	5.21	4.55	5.04	5.06	5.09	5.05	4.51	4.27	4.86	4.80	4.82	4.50
Sub. G	100 m	7.18	6.94	7.20	6.79	6.67	6.47	6.43	6.66	6.23	6.51	6.36	6.00	5.95	6.47	5.67	5.94	5.71
Sub. H	100 m	6.32	6.41	6.00	5.84	5.79	6.00	5.50	5.57	5.31	5.63	5.61	5.73	5.28	5.17	5.16	5.29	5.37
Sub. I	100 m	5.96	6.98	6.22	6.54	6.16	6.36	5.62	6.07	6.08	5.90	5.79	5.71	5.38	5.74	5.63	5.51	5.45

		6.0 m/s					6.5 m/s				7.0 m/s			7.5 m/s		8.0 m/s	8.5 m/s	9.0 m/s
		Load (kg)					Load (kg)				Load (kg)			Load (kg)		Load (kg)	Load (kg)	Load (kg)
	Specialty	0	4	6	8	10	0	4	6	8	0	4	6	0	4	0	0	0
Sub. A	100 m	3.50	3.49	3.97	3.60	3.52	3.21	3.29	3.60	3.30	2.93	3.03	3.27	2.83	2.62	2.24	1.62	1.09
Sub. B	100 m	4.55	4.62	4.69	4.86	4.80	4.19	4.30	4.23	4.34	3.74	3.76	3.77	3.37	3.30	2.82	2.36	1.77
Sub. C	200m, 400 m	4.78	4.75	4.83	4.58	4.69	4.42	4.18	4.40	4.24	4.13	4.01	4.03	3.63	3.46	3.06	2.41	1.98
Sub. D	100 m	5.42	5.26	5.49	5.55	5.18	4.83	4.96	4.96	4.98	4.23	4.61	4.53	3.94	4.15	3.49	2.98	2.64
Sub.E	100 m	4.48	4.31	4.54	4.06	4.16	4.25	3.91	4.08	4.01	3.79	3.65	3.47	3.24	3.28	3.00	2.45	2.01
Sub. F	100 m	4.39	4.18	4.58	4.53	4.19	4.07	4.14	4.09	4.01	3.52	3.79	3.82	3.10	3.31	2.93	2.53	2.16
Sub. G	100 m	5.51	5.56	6.11	5.54	5.38	5.43	5.26	5.48	5.37	5.14	4.97	4.81	4.67	4.25	4.08	3.56	2.99
Sub. H	100 m	5.42	5.29	5.01	5.07	4.99	5.18	4.79	4.68	4.77	4.68	4.39	4.42	4.26	4.14	4.00	3.61	3.16
Sub. I	100 m	5.39	4.94	5.47	5.35	5.24	5.07	4.52	4.86	4.79	4.65	4.20	4.43	4.15	3.86	3.53	3.21	2.44

Individual values of step-averaged vertical ground reaction force (N/kg)

		4.0 m/s		4.5 m/s				5.0 m/s					5.5 m/s					
		Load (kg)		Load (kg)				Load (kg)					Load (kg)					
	Specialty	10	12	6	8	10	12	4	6	8	10	12	0	4	6	8	10	12
Sub. A	100 m	9.98	9.90	9.82	9.83	9.98	9.79	9.72	9.45	10.09	9.79	9.93	9.61	9.63	9.76	9.93	9.61	9.70
Sub. B	100 m	9.58	9.73	9.49	9.94	10.07	9.86	9.68	10.28	10.00	10.23	9.77	9.39	10.34	9.99	9.69	9.64	9.81
Sub. C	200m, 400 m	9.63	9.76	9.49	9.80	9.52	9.78	9.60	9.61	9.76	9.95	10.03	9.55	9.61	9.87	9.70	9.67	9.56
Sub. D	100 m	9.81	9.43	10.46	10.05	10.03	9.92	9.86	9.95	9.96	9.91	10.33	9.52	10.33	9.64	9.96	9.82	9.79
Sub.E	100 m	9.62	10.08	9.99	9.49	9.85	10.02	9.98	9.90	9.80	10.18	9.81	9.79	10.00	9.77	10.06	9.85	9.81
Sub. F	100 m	9.69	10.06	9.65	9.97	10.14	10.39	9.91	10.17	9.84	9.87	9.90	9.66	10.23	10.01	9.90	9.55	9.80
Sub. G	100 m	9.66	9.92	10.01	10.05	9.79	9.87	9.88	9.90	10.25	9.67	9.46	9.99	10.13	9.67	10.27	9.76	9.87
Sub. H	100 m	10.19	10.24	9.79	10.21	10.04	9.86	10.33	9.91	10.56	9.88	9.93	9.67	10.00	10.01	9.80	9.96	9.83
Sub. I	100 m	9.97	9.52	9.82	9.87	9.88	10.00	9.51	9.78	9.88	9.88	10.19	9.30	9.66	9.74	9.86	9.88	9.72

		6.0 m/s					6.5 m/s				7.0 m/s			7.5 m/s		8.0 m/s	8.5 m/s	9.0 m/s
		Load (kg)					Load (kg)				Load (kg)			Load (kg)		Load (kg)	Load (kg)	Load (kg)
	Specialty	0	4	6	8	10	0	4	6	8	0	4	6	0	4	0	0	0
Sub. A	100 m	9.69	9.78	9.85	9.58	9.81	9.67	9.74	9.75	9.55	9.68	9.58	9.47	9.47	9.53	9.46	9.61	9.52
Sub. B	100 m	9.94	9.59	9.54	9.78	9.67	9.78	9.53	9.76	9.57	9.45	9.62	9.51	9.46	9.51	9.55	9.37	9.58
Sub. C	200m, 400 m	9.83	9.66	9.73	9.76	9.44	9.51	9.76	9.54	9.44	9.20	9.43	9.50	9.28	9.34	9.45	9.27	9.40
Sub. D	100 m	9.75	10.50	10.27	10.08	9.89	9.97	9.98	10.23	10.26	9.88	9.69	9.80	9.82	10.02	10.00	9.66	9.72
Sub.E	100 m	9.77	9.65	9.68	9.73	9.80	9.39	9.64	9.55	9.50	9.59	9.83	9.74	9.53	9.57	9.53	9.50	9.60
Sub. F	100 m	9.83	10.08	9.58	9.76	9.84	9.79	9.36	9.79	9.72	9.59	9.54	9.57	9.60	9.61	9.67	9.39	9.56
Sub. G	100 m	9.98	10.05	9.82	9.69	9.51	9.55	9.73	9.95	9.58	9.54	9.62	9.51	9.62	9.62	9.56	9.61	9.59
Sub. H	100 m	9.66	9.42	9.63	9.70	9.76	9.66	9.94	9.76	9.80	9.69	10.00	9.80	9.58	9.57	9.55	9.63	9.63
Sub. I	100 m	9.49	9.78	9.66	9.66	9.72	9.48	9.79	9.71	9.78	9.48	9.75	9.66	9.52	9.43	9.55	9.35	9.49

Individual values of stance-averaged vertical ground reaction force (N/kg)

		4.0 m/s		4.5 m/s				5.0 m/s					5.5 m/s					
		Load (kg)		Load (kg)				Load (kg)					Load (kg)					
	Specialty	10	12	6	8	10	12	4	6	8	10	12	0	4	6	8	10	12
Sub. A	100 m	12.22	12.40	13.45	11.73	12.82	12.39	12.39	13.86	12.15	13.24	13.32	12.75	12.67	14.05	12.99	13.72	14.18
Sub. B	100 m	13.24	14.10	13.10	13.36	13.58	14.07	13.57	13.53	14.15	14.02	14.37	14.27	14.73	14.34	14.98	15.15	14.86
Sub. C	200m, 400 m	12.53	11.55	13.64	13.08	13.41	12.11	13.36	14.07	13.64	13.83	13.16	14.66	14.03	14.42	14.56	14.80	14.46
Sub. D	100 m	12.64	12.15	13.36	13.48	13.10	12.40	14.15	13.97	14.45	13.78	12.69	14.14	14.48	14.68	14.78	14.53	13.68
Sub.E	100 m	12.81	11.90	13.35	12.83	13.17	12.53	13.03	14.13	13.06	13.34	13.62	14.49	13.78	14.76	13.71	13.81	14.31
Sub. F	100 m	11.99	11.64	11.95	11.97	12.59	12.10	11.56	12.98	13.16	13.38	12.94	12.27	12.13	14.09	13.92	13.50	13.40
Sub. G	100 m	13.92	12.54	14.75	13.31	14.26	13.98	14.45	15.75	14.00	14.68	14.60	15.44	15.18	16.34	14.92	15.37	15.24
Sub. H	100 m	12.81	12.90	12.86	12.94	13.18	13.75	13.19	13.72	13.61	14.42	14.77	14.26	13.93	14.67	14.58	15.05	15.23
Sub. I	100 m	11.86	13.61	13.74	14.82	13.60	13.89	13.74	15.91	15.64	15.39	14.61	15.92	14.69	17.14	16.62	16.26	15.47

		6.0 m/s					6.5 m/s				7.0 m/s			7.5 m/s		8.0 m/s	8.5 m/s	9.0 m/s
		Load (kg)					Load (kg)				Load (kg)			Load (kg)		Load (kg)	Load (kg)	Load (kg)
	Specialty	0	4	6	8	10	0	4	6	8	0	4	6	0	4	0	0	0
Sub. A	100 m	13.41	13.48	14.63	13.91	14.53	13.96	14.41	15.66	14.52	15.20	15.84	16.76	16.28	16.76	16.93	17.68	19.77
Sub. B	100 m	14.85	15.22	14.97	15.98	15.90	15.19	15.74	16.33	16.40	15.77	16.36	17.02	16.43	16.79	17.22	18.33	19.05
Sub. C	200m, 400 m	15.49	14.69	15.33	15.36	15.59	16.14	15.14	16.33	16.15	16.59	16.67	16.93	17.29	17.26	18.13	18.62	19.51
Sub. D	100 m	14.71	15.03	15.38	15.18	15.23	15.05	15.88	15.89	16.33	15.14	16.66	16.54	16.00	17.58	17.24	17.84	19.22
Sub.E	100 m	14.89	15.03	15.70	14.65	15.46	16.00	15.94	16.41	16.08	17.07	17.26	16.77	17.71	18.24	18.43	19.23	20.52
Sub. F	100 m	12.96	13.97	14.45	14.49	14.53	13.76	15.28	15.52	15.55	14.71	15.56	16.52	15.57	16.69	17.06	18.36	19.14
Sub. G	100 m	15.92	15.93	16.93	16.35	15.50	16.72	16.96	17.44	16.95	17.40	17.37	17.51	17.92	17.45	18.44	19.06	19.93
Sub. H	100 m	14.96	14.63	15.17	15.73	15.85	15.84	15.39	15.81	16.65	16.58	15.92	16.62	17.14	16.86	17.72	18.56	19.32
Sub. I	100 m	16.89	15.29	17.77	17.33	17.21	17.68	16.03	18.27	18.42	18.22	17.25	18.82	18.62	18.88	19.61	20.37	20.72

Individual values of net anteroposterior impulse (Ns/kg)

		4.0 m/s		4.5 m/s				5.0 m/s					5.5 m/s					
		Load (kg)		Load (kg)				Load (kg)					Load (kg)					
	Specialty	10	12	6	8	10	12	4	6	8	10	12	0	4	6	8	10	12
Sub. A	100 m	1.00	1.03	0.86	0.92	0.91	0.90	0.81	0.74	0.83	0.78	0.75	0.66	0.68	0.67	0.68	0.67	0.63
Sub. B	100 m	1.10	1.03	1.01	0.96	1.01	0.96	0.84	0.85	0.85	0.87	0.86	0.66	0.73	0.69	0.75	0.73	0.78
Sub. C	200m, 400 m	1.05	1.08	0.93	0.94	0.95	0.96	0.87	0.83	0.84	0.88	0.85	0.74	0.78	0.76	0.72	0.69	0.69
Sub. D	100 m	1.33	1.32	1.19	1.18	1.15	1.16	1.05	1.00	1.01	1.05	1.04	0.96	0.95	0.90	0.91	0.94	0.85
Sub.E	100 m	1.22	1.23	1.05	1.06	1.07	1.00	0.89	0.92	0.92	0.96	0.86	0.77	0.78	0.80	0.77	0.76	0.77
Sub. F	100 m	1.13	1.18	1.02	1.00	1.02	1.05	0.90	0.88	0.87	0.87	0.89	0.81	0.80	0.78	0.78	0.78	0.76
Sub. G	100 m	1.24	1.29	1.17	1.21	1.10	1.10	1.05	1.00	1.06	1.01	0.98	0.96	0.92	0.90	0.89	0.89	0.87
Sub. H	100 m	1.16	1.19	1.03	1.02	0.98	0.97	0.94	0.87	0.90	0.85	0.84	0.83	0.81	0.77	0.76	0.77	0.77
Sub. I	100 m	1.18	1.13	1.00	0.99	1.02	1.05	0.90	0.83	0.87	0.84	0.92	0.72	0.79	0.73	0.77	0.74	0.76

		6.0 m/s					6.5 m/s				7.0 m/s			7.5 m/s		8.0 m/s	8.5 m/s	9.0 m/s
		Load (kg)					Load (kg)				Load (kg)			Load (kg)		Load (kg)	Load (kg)	Load (kg)
	Specialty	0	4	6	8	10	0	4	6	8	0	4	6	0	4	0	0	0
Sub. A	100 m	0.55	0.56	0.58	0.56	0.54	0.48	0.49	0.50	0.49	0.41	0.40	0.41	0.36	0.33	0.27	0.19	0.12
Sub. B	100 m	0.63	0.63	0.64	0.64	0.63	0.56	0.57	0.54	0.54	0.47	0.46	0.45	0.40	0.39	0.32	0.24	0.18
Sub. C	200m, 400 m	0.65	0.68	0.67	0.63	0.61	0.56	0.58	0.56	0.53	0.49	0.48	0.49	0.41	0.40	0.33	0.25	0.20
Sub. D	100 m	0.86	0.83	0.83	0.83	0.82	0.74	0.70	0.73	0.71	0.63	0.60	0.61	0.55	0.53	0.46	0.36	0.30
Sub.E	100 m	0.68	0.65	0.67	0.64	0.63	0.58	0.55	0.56	0.57	0.49	0.48	0.48	0.40	0.40	0.36	0.28	0.22
Sub. F	100 m	0.74	0.68	0.68	0.70	0.65	0.65	0.58	0.58	0.57	0.52	0.53	0.50	0.43	0.43	0.37	0.29	0.24
Sub. G	100 m	0.83	0.81	0.82	0.76	0.77	0.74	0.70	0.73	0.69	0.65	0.63	0.61	0.57	0.53	0.48	0.41	0.33
Sub. H	100 m	0.73	0.72	0.69	0.68	0.68	0.66	0.65	0.63	0.61	0.57	0.59	0.56	0.50	0.51	0.45	0.39	0.33
Sub. I	100 m	0.65	0.70	0.66	0.66	0.66	0.58	0.61	0.56	0.56	0.52	0.52	0.50	0.45	0.42	0.36	0.31	0.23

Individual values of net vertical impulse (Ns/kg)

		4.0 m/s		4.5 m/s				5.0 m/s					5.5 m/s					
		Load (kg)		Load (kg)				Load (kg)					Load (kg)					
	Specialty	10	12	6	8	10	12	4	6	8	10	12	0	4	6	8	10	12
Sub. A	100 m	2.27	2.29	2.17	2.19	2.24	2.20	2.11	2.07	2.23	2.22	2.22	2.08	2.09	2.14	2.20	2.21	2.24
Sub. B	100 m	2.13	2.10	2.16	2.16	2.16	2.15	2.04	2.22	2.15	2.17	2.16	1.95	2.17	2.13	2.08	2.10	2.15
Sub. C	200m, 400 m	2.16	2.21	2.15	2.19	2.08	2.14	2.14	2.16	2.12	2.19	2.17	2.08	2.09	2.17	2.08	2.09	2.06
Sub. D	100 m	2.56	2.42	2.59	2.45	2.54	2.41	2.32	2.34	2.32	2.49	2.44	2.33	2.37	2.20	2.27	2.42	2.27
Sub.E	100 m	2.59	2.59	2.51	2.39	2.53	2.49	2.42	2.41	2.42	2.49	2.34	2.30	2.32	2.32	2.46	2.36	2.34
Sub. F	100 m	2.33	2.40	2.25	2.31	2.37	2.46	2.27	2.28	2.26	2.29	2.29	2.19	2.27	2.25	2.27	2.21	2.26
Sub. G	100 m	2.42	2.36	2.38	2.37	2.36	2.37	2.34	2.34	2.37	2.29	2.25	2.47	2.35	2.26	2.33	2.31	2.31
Sub. H	100 m	2.35	2.39	2.21	2.26	2.22	2.22	2.25	2.14	2.31	2.18	2.22	2.06	2.14	2.18	2.14	2.19	2.19
Sub. I	100 m	2.34	2.23	2.20	2.24	2.22	2.32	2.18	2.18	2.24	2.20	2.32	2.01	2.17	2.18	2.26	2.19	2.17

		6.0 m/s					6.5 m/s				7.0 m/s			7.5 m/s		8.0 m/s	8.5 m/s	9.0 m/s
		Load (kg)					Load (kg)				Load (kg)			Load (kg)		Load (kg)	Load (kg)	Load (kg)
	Specialty	0	4	6	8	10	0	4	6	8	0	4	6	0	4	0	0	0
Sub. A	100 m	2.11	2.16	2.14	2.16	2.25	2.10	2.13	2.17	2.17	2.12	2.11	2.13	2.09	2.13	2.08	2.11	2.12
Sub. B	100 m	2.06	2.09	2.06	2.11	2.08	2.03	2.07	2.09	2.05	1.97	2.01	2.03	1.96	1.97	1.94	1.90	1.93
Sub. C	200m, 400 m	2.11	2.11	2.14	2.10	2.03	2.04	2.10	2.07	2.02	1.96	2.01	2.04	1.96	2.00	1.98	1.94	1.95
Sub. D	100 m	2.31	2.37	2.33	2.27	2.40	2.32	2.24	2.33	2.32	2.26	2.17	2.24	2.23	2.24	2.25	2.16	2.16
Sub.E	100 m	2.26	2.26	2.31	2.33	2.34	2.16	2.23	2.26	2.30	2.19	2.28	2.31	2.21	2.25	2.19	2.17	2.21
Sub. F	100 m	2.19	2.25	2.15	2.23	2.27	2.18	2.14	2.21	2.22	2.16	2.17	2.16	2.15	2.16	2.17	2.09	2.14
Sub. G	100 m	2.39	2.32	2.28	2.23	2.22	2.26	2.26	2.31	2.19	2.21	2.22	2.21	2.19	2.20	2.19	2.19	2.19
Sub. H	100 m	2.01	2.00	2.09	2.12	2.18	2.02	2.10	2.12	2.15	2.03	2.14	2.12	2.01	2.06	2.00	2.01	2.00
Sub. I	100 m	2.04	2.18	2.14	2.16	2.17	2.03	2.15	2.12	2.15	2.02	2.12	2.11	2.01	2.05	2.01	1.95	1.93

Individual values of propulsive impulse (Ns/kg)

		4.0 m/s		4.5 m/s				5.0 m/s					5.5 m/s					
		Load (kg)		Load (kg)				Load (kg)					Load (kg)					
	Specialty	10	12	6	8	10	12	4	6	8	10	12	0	4	6	8	10	12
Sub. A	100 m	1.05	1.07	0.90	0.98	0.97	0.95	0.87	0.79	0.91	0.85	0.82	0.75	0.76	0.74	0.76	0.74	0.70
Sub. B	100 m	1.10	1.03	1.02	0.97	1.01	0.96	0.85	0.87	0.86	0.90	0.86	0.67	0.75	0.72	0.76	0.74	0.79
Sub. C	200m, 400 m	1.05	1.09	0.93	0.95	0.95	0.98	0.87	0.84	0.85	0.90	0.88	0.75	0.80	0.78	0.75	0.72	0.72
Sub. D	100 m	1.34	1.32	1.20	1.19	1.16	1.17	1.07	1.01	1.03	1.07	1.07	0.97	0.97	0.91	0.92	0.95	0.89
Sub.E	100 m	1.23	1.27	1.06	1.07	1.08	1.03	0.92	0.93	0.94	0.98	0.88	0.81	0.81	0.82	0.80	0.81	0.81
Sub. F	100 m	1.13	1.19	1.03	1.00	1.02	1.07	0.90	0.90	0.88	0.89	0.91	0.84	0.82	0.80	0.80	0.80	0.78
Sub. G	100 m	1.24	1.29	1.18	1.22	1.10	1.10	1.06	1.00	1.07	1.01	0.98	0.97	0.93	0.90	0.91	0.90	0.88
Sub. H	100 m	1.16	1.19	1.03	1.02	0.98	0.98	0.97	0.88	0.92	0.87	0.86	0.84	0.83	0.79	0.78	0.79	0.79
Sub. I	100 m	1.20	1.14	1.02	1.01	1.04	1.07	0.92	0.86	0.90	0.88	0.96	0.75	0.83	0.77	0.80	0.78	0.81

		6.0 m/s					6.5 m/s				7.0 m/s			7.5 m/s		8.0 m/s	8.5 m/s	9.0 m/s
		Load (kg)					Load (kg)				Load (kg)			Load (kg)		Load (kg)	Load (kg)	Load (kg)
	Specialty	0	4	6	8	10	0	4	6	8	0	4	6	0	4	0	0	0
Sub. A	100 m	0.65	0.65	0.66	0.64	0.62	0.58	0.57	0.57	0.56	0.49	0.49	0.49	0.43	0.42	0.37	0.31	0.26
Sub. B	100 m	0.64	0.65	0.65	0.66	0.64	0.58	0.58	0.55	0.56	0.49	0.48	0.48	0.44	0.43	0.37	0.32	0.30
Sub. C	200m, 400 m	0.68	0.72	0.69	0.66	0.65	0.60	0.63	0.59	0.58	0.54	0.53	0.54	0.47	0.46	0.41	0.35	0.32
Sub. D	100 m	0.87	0.85	0.85	0.84	0.84	0.77	0.73	0.75	0.73	0.65	0.63	0.65	0.57	0.57	0.50	0.43	0.40
Sub.E	100 m	0.71	0.69	0.70	0.69	0.67	0.62	0.59	0.60	0.61	0.54	0.53	0.53	0.46	0.46	0.42	0.36	0.33
Sub. F	100 m	0.77	0.71	0.70	0.72	0.68	0.68	0.61	0.61	0.60	0.57	0.55	0.54	0.49	0.48	0.45	0.39	0.36
Sub. G	100 m	0.84	0.83	0.83	0.77	0.78	0.75	0.73	0.74	0.71	0.68	0.66	0.64	0.60	0.58	0.53	0.47	0.40
Sub. H	100 m	0.75	0.74	0.71	0.71	0.71	0.69	0.68	0.65	0.65	0.61	0.62	0.60	0.55	0.55	0.51	0.46	0.41
Sub. I	100 m	0.69	0.75	0.71	0.71	0.70	0.63	0.65	0.62	0.62	0.57	0.57	0.55	0.51	0.48	0.44	0.39	0.34

Individual values of braking impulse (Ns/kg)

		4.0 m/s		4.5 m/s				5.0 m/s					5.5 m/s					
		Load (kg)		Load (kg)				Load (kg)					Load (kg)					
	Specialty	10	12	6	8	10	12	4	6	8	10	12	0	4	6	8	10	12
Sub. A	100 m	-0.05	-0.04	-0.04	-0.05	-0.06	-0.05	-0.06	-0.05	-0.08	-0.07	-0.07	-0.09	-0.08	-0.07	-0.09	-0.07	-0.07
Sub. B	100 m	0.00	-0.01	-0.01	-0.01	0.00	-0.01	-0.01	-0.02	-0.01	-0.02	-0.01	-0.01	-0.02	-0.02	-0.01	-0.01	-0.01
Sub. C	200m, 400 m	0.00	-0.01	0.00	-0.01	-0.01	-0.02	0.00	-0.01	-0.02	-0.02	-0.03	-0.01	-0.02	-0.02	-0.03	-0.03	-0.03
Sub. D	100 m	-0.01	0.00	-0.01	-0.01	-0.02	-0.01	-0.02	-0.01	-0.01	-0.01	-0.02	-0.01	-0.02	-0.01	-0.01	-0.01	-0.04
Sub.E	100 m	-0.01	-0.04	-0.01	-0.01	-0.01	-0.04	-0.03	-0.01	-0.02	-0.02	-0.03	-0.03	-0.04	-0.02	-0.04	-0.04	-0.04
Sub. F	100 m	0.00	-0.01	-0.01	-0.01	0.00	-0.02	0.00	-0.02	-0.01	-0.02	-0.02	-0.03	-0.01	-0.02	-0.02	-0.02	-0.02
Sub. G	100 m	-0.01	0.00	-0.01	0.00	0.00	0.00	-0.01	-0.01	-0.02	0.00	0.00	-0.01	-0.01	0.00	-0.02	-0.01	-0.01
Sub. H	100 m	0.00	0.00	0.00	-0.01	0.00	-0.01	-0.03	-0.01	-0.02	-0.01	-0.02	-0.01	-0.02	-0.02	-0.02	-0.02	-0.02
Sub. I	100 m	-0.02	-0.01	-0.01	-0.02	-0.03	-0.02	-0.02	-0.03	-0.03	-0.04	-0.04	-0.03	-0.04	-0.04	-0.04	-0.04	-0.05

		6.0 m/s					6.5 m/s				7.0 m/s			7.5 m/s		8.0 m/s	8.5 m/s	9.0 m/s
		Load (kg)					Load (kg)				Load (kg)			Load (kg)		Load (kg)	Load (kg)	Load (kg)
	Specialty	0	4	6	8	10	0	4	6	8	0	4	6	0	4	0	0	0
Sub. A	100 m	-0.10	-0.09	-0.08	-0.08	-0.07	-0.09	-0.08	-0.08	-0.07	-0.08	-0.08	-0.07	-0.07	-0.09	-0.10	-0.11	-0.14
Sub. B	100 m	-0.01	-0.01	-0.01	-0.01	-0.01	-0.02	-0.01	-0.01	-0.02	-0.02	-0.02	-0.03	-0.03	-0.04	-0.05	-0.08	-0.12
Sub. C	200m, 400 m	-0.03	-0.04	-0.02	-0.04	-0.04	-0.04	-0.05	-0.04	-0.05	-0.05	-0.05	-0.05	-0.06	-0.05	-0.08	-0.10	-0.12
Sub. D	100 m	-0.02	-0.02	-0.02	-0.01	-0.02	-0.02	-0.03	-0.03	-0.02	-0.02	-0.03	-0.03	-0.02	-0.04	-0.04	-0.07	-0.10
Sub.E	100 m	-0.03	-0.04	-0.03	-0.04	-0.04	-0.04	-0.04	-0.04	-0.04	-0.05	-0.05	-0.05	-0.06	-0.06	-0.06	-0.08	-0.11
Sub. F	100 m	-0.03	-0.03	-0.02	-0.02	-0.02	-0.04	-0.03	-0.03	-0.03	-0.05	-0.03	-0.04	-0.06	-0.05	-0.08	-0.11	-0.12
Sub. G	100 m	-0.01	-0.02	-0.01	-0.01	-0.01	-0.02	-0.02	-0.01	-0.02	-0.02	-0.02	-0.03	-0.03	-0.04	-0.04	-0.06	-0.07
Sub. H	100 m	-0.02	-0.02	-0.02	-0.03	-0.02	-0.03	-0.03	-0.02	-0.03	-0.04	-0.04	-0.03	-0.05	-0.05	-0.06	-0.07	-0.09
Sub. I	100 m	-0.04	-0.04	-0.05	-0.04	-0.04	-0.05	-0.05	-0.05	-0.06	-0.06	-0.05	-0.06	-0.06	-0.06	-0.08	-0.09	-0.11

Individual values of RF

	Specialty	4.0 m/s Load (kg) 10	4.0 m/s 12	4.5 m/s Load (kg) 6	4.5 m/s 8	4.5 m/s 10	4.5 m/s 12	5.0 m/s Load (kg) 4	5.0 m/s 6	5.0 m/s 8	5.0 m/s 10	5.0 m/s 12	5.5 m/s Load (kg) 0	5.5 m/s 4	5.5 m/s 6	5.5 m/s 8	5.5 m/s 10	5.5 m/s 12
Sub. A	100 m	0.393	0.401	0.358	0.378	0.366	0.369	0.344	0.327	0.337	0.319	0.313	0.288	0.296	0.291	0.281	0.280	0.264
Sub. B	100 m	0.453	0.434	0.418	0.404	0.418	0.401	0.376	0.348	0.360	0.366	0.364	0.318	0.311	0.301	0.331	0.323	0.333
Sub. C	200m, 400 m	0.432	0.433	0.391	0.391	0.408	0.404	0.370	0.352	0.361	0.367	0.355	0.331	0.342	0.322	0.319	0.307	0.308
Sub. D	100 m	0.456	0.473	0.412	0.427	0.406	0.428	0.409	0.389	0.392	0.383	0.383	0.375	0.364	0.370	0.362	0.355	0.340
Sub.E	100 m	0.418	0.418	0.379	0.401	0.384	0.364	0.339	0.353	0.350	0.351	0.336	0.312	0.310	0.320	0.290	0.299	0.306
Sub. F	100 m	0.433	0.436	0.412	0.390	0.391	0.389	0.361	0.357	0.353	0.349	0.356	0.341	0.325	0.319	0.319	0.328	0.313
Sub. G	100 m	0.452	0.480	0.432	0.449	0.418	0.416	0.402	0.385	0.401	0.400	0.393	0.356	0.359	0.363	0.348	0.355	0.345
Sub. H	100 m	0.435	0.441	0.420	0.408	0.398	0.395	0.375	0.374	0.357	0.358	0.348	0.366	0.345	0.325	0.326	0.326	0.325
Sub. I	100 m	0.442	0.448	0.410	0.397	0.407	0.407	0.373	0.352	0.354	0.350	0.360	0.332	0.336	0.310	0.314	0.314	0.322

	Specialty	6.0 m/s Load (kg) 0	6.0 m/s 4	6.0 m/s 6	6.0 m/s 8	6.0 m/s 10	6.5 m/s Load (kg) 0	6.5 m/s 4	6.5 m/s 6	6.5 m/s 8	7.0 m/s Load (kg) 0	7.0 m/s 4	7.0 m/s 6	7.5 m/s Load (kg) 0	7.5 m/s 4	8.0 m/s Load (kg) 0	8.5 m/s Load (kg) 0	9.0 m/s Load (kg) 0
Sub. A	100 m	0.240	0.240	0.252	0.242	0.228	0.214	0.214	0.216	0.214	0.182	0.181	0.185	0.166	0.149	0.127	0.088	0.053
Sub. B	100 m	0.285	0.284	0.292	0.284	0.283	0.258	0.257	0.245	0.249	0.224	0.218	0.210	0.195	0.187	0.156	0.123	0.089
Sub. C	200m, 400 m	0.287	0.298	0.293	0.277	0.279	0.256	0.257	0.252	0.246	0.233	0.225	0.223	0.197	0.189	0.159	0.123	0.096
Sub. D	100 m	0.340	0.322	0.328	0.336	0.315	0.296	0.291	0.289	0.285	0.261	0.261	0.257	0.233	0.223	0.192	0.159	0.131
Sub.E	100 m	0.281	0.268	0.270	0.259	0.253	0.250	0.231	0.234	0.236	0.209	0.200	0.196	0.174	0.171	0.156	0.122	0.094
Sub. F	100 m	0.313	0.280	0.295	0.293	0.271	0.276	0.255	0.249	0.243	0.226	0.230	0.219	0.189	0.189	0.163	0.131	0.107
Sub. G	100 m	0.322	0.323	0.335	0.314	0.321	0.302	0.290	0.295	0.296	0.277	0.268	0.259	0.245	0.229	0.209	0.178	0.144
Sub. H	100 m	0.333	0.330	0.305	0.299	0.293	0.301	0.288	0.276	0.268	0.263	0.256	0.249	0.232	0.229	0.212	0.182	0.154
Sub. I	100 m	0.296	0.299	0.287	0.287	0.284	0.268	0.264	0.249	0.245	0.240	0.230	0.222	0.210	0.194	0.171	0.150	0.112
